# Editorial: Women in psychiatry 2021: Autism

**DOI:** 10.3389/fpsyt.2022.1090395

**Published:** 2022-12-15

**Authors:** Rita Barone, Costanza Colombi

**Affiliations:** ^1^Child Neuropsychiatry Unit, Department of Clinical and Experimental Medicine, University of Catania, Catania, Italy; ^2^IRCCS Fondazione Stella Maris, Pisa, Italy

**Keywords:** autism, diagnosis, comorbidities, patho-mechanisms, interventions

Autism Spectrum Disorder (ASD) is a neurodevelopmental disturbance characterized by impairment in reciprocal social interaction and communication and repetitive, stereotyped patterns of behaviors, interests, and activities. Autism core features frequently associate with co-occurring difficulties or disorders, which greatly influence independence and wellbeing. As such, ASD represents a lifelong condition harboring a significant burden for patients and their families.

This challenging Research Topic was contributed by women scientists working in the field of ASD. Seven articles on most debated autism topics with contributions of researchers from all over the world were collected. Hereby, we will highlight relevant results and conclusions from all the articles published in the Research Topic including studies on ASD diagnosis, comorbidities, molecular mechanisms, developmental trajectories and interventions.

## Diagnosis and early detection

To date, gold standard tools for ASD diagnosis are based on direct observation of behavior (Autism Diagnostic Observations Schedule, ADOS) and an anamnestic parents' interview (Autism Diagnostic Interview Revised, ADI-R). In this Research Topic, two studies pursued novel approaches to prompt ASD diagnosis. Kamp-Becker et al. applied Random Forest classifier to detect those ADOS and ADI-R items sufficient to differentiate ASD from other mental disorders or developmental disorders in a large sample of subjects aged from 4 to 72 years (*N* = 2310). Modeling was performed for two subsamples of children/young adolescents and adolescents/adults. This elegant work shows that classifiers built on a reduced number of items (8–11) mainly based on ADOS yield satisfactory sensitivity and specificity values pursuing the way to reduce the complexity of diagnostic procedures.

Among measures for early detection, the Rapid Interactive screening Test for Autism in Toddlers (RITA-T) is a screening measure for ASD in children 18–36 months old. Kong et al. applied the RITA-T to a sample of children aged 18–84 months at risk for ASD. In particular, the RITA-T was used in a larger age range than originally reported also including individuals from different ethnic groups. The study found a sensitivity of 81% and a specificity of 89% in the whole group (*N* = 35) and significant correlations between RITA-T and ADOS-2 scores supporting the potential usefulness of the RITA-T as early screening method for ASD.

## Comorbidities

Previous studies showed that adults with ASD might be particularly vulnerable to the impact of COVID-19 pandemic albeit with varying severity. Halstead et al. evaluated the impact of COVID-19 lockdown on sleep disturbances in adults with ASD. Comorbid sleep problems pre-existing to the pandemic were highly represented in the study sample (*N* = 95). The study shows that sleep latency and duration and daytime dysfunction scores on the Pittsburgh Sleep Quality Index (PSQI) improved significantly during the lockdown. According to the Authors, positive outcomes might be related to the decrease in social demands and sensory inputs caused by quarantine in people with ASD. Mutluer et al. systematically reviewed studies focused on population-analyses for rating the psychiatric comorbidities in children and adolescents with ASD. Thirty-nine articles published between 2015 and 2020 were included. Reported prevalence figures were generally lower than those computed in clinical or treatment samples usually collecting complex patients with higher number of comorbidities. Moreover, rates of comorbidities in population-studies were found to be highly heterogeneous reflecting diversity of diagnostic tools used for both ASD and comorbidities.

## Molecular mechanisms

As to molecular mechanisms of ASD,  McLellan et al. reviewed their own and additional studies on maternal autoantibody related autism (MAR-ASD), a condition accounting for up to 20% of ASD. MAR-ASD is characterized by maternal autoantibody production, and skewed maternal and fetal chemokine/cytokine profile. To understand the role of maternal autoantibodies in neurodevelopment, animal models including mice, rats, and non-human primates have been used for passive transfer studies. Recently, using antigen driven mouse model, researchers were able to test the behavioral and neurological impacts of constant gestational exposure to maternal autoantibodies. Interestingly, in both instances, there was an increase of brain volume in the offspring from treated dams thus suggesting relationships between maternal immune dysregulation during gestation and changes in the brain size of affected offspring.

## Developmental trajectories and outcomes

By using a well-designed analyses, Landa et al. prospectively scanned ASD developmental trajectories and their predictors. Children with elevated likelihood for ASD (ELA) (younger siblings of children with ASD, *N* = 210) were examined in three time points. Stability of diagnosis was evaluated at the last time-point (school-age). Some children showed a stable diagnosis whereas others had a dynamic pattern of classification over time. The “lost diagnosis” group (*n* = 23) met ASD criteria at time 1 and/or at time 2, but not at time 3. The “later diagnosed” group met diagnosis at time 3 but not at time 1 and/or time 2 indicating the importance to extend surveillance for ASD to at least middle childhood. A greatest proportion of instability of diagnostic classification was detected between the first (mean age of 15 months) and second examination (36 months of age) with almost 85% of “later diagnosed” cases being diagnosed by age 3 years. Finally, the study found that the “lost-diagnosis” group had significant gain in verbal IQ and reduction in ASD symptom severity from age 15 months to school-age than the “later diagnosed” group.

## Interventions

Pervin et al. conducted a meta-review comparing interventions for ASD in high-income countries (HIC) and in lower-middle or lower-upper income countries (LMIC and LUIC, respectively). Thirty-five systematic reviews (2011–2021), were included. This meta-review illustrates that the majority of studies from LMIC were not providing sufficient power to be considered evidence-based. Moreover, the study shows that examined systematic reviews did not focus on comparisons for intervention effectiveness between HIC and LMIC. Further efforts for spreading evidence-based models for interventions in LMIC are urgently required.

In conclusion, various studies from this Research Topic focused on some unsolved questions in ASD research such as early screening methods and diagnostic procedures implementations, comorbidity prevalence rates in ASD population as well as to prospectively analyze the ASD developmental trajectories. Moreover, ensuring access to evidence-based interventions in low-income and developing countries represents a crucial point to be particularly envisaged.

## Author contributions

Both authors listed have made a substantial, direct, and intellectual contribution to the work and approved it for publication.

